# Process evaluation of the Bridging the Age Gap in Breast Cancer decision support intervention cluster randomised trial

**DOI:** 10.1186/s13063-021-05360-z

**Published:** 2021-07-13

**Authors:** Maria Burton, Kate J. Lifford, Lynda Wyld, Fiona Armitage, Alistair Ring, Anthony Nettleship, Karen Collins, Jenna Morgan, Malcolm W. R. Reed, Geoffrey R. Holmes, Mike Bradburn, Jacqui Gath, Tracy Green, Deirdre Revell, Kate Brain, Adrian Edwards, On behalf of the Study Management Team, Helena Harder, Susan Ward, Paul Richards, Charlene Martin, Tim Chater, Kirsty Pemberton, Christopher Murray, Stephen Walters, Esther Herbert, Thompson Robinson, Kwok Leung Cheung, Riccardo Audisio

**Affiliations:** 1 College of Health, Wellbeing & Life Sciences, Sheffield Hallam University, Collegiate Crescent, Sheffield, UK; 2 Division of Population Medicine, School of Medicine, Cardiff University, Neuadd Meirionnydd, Heath Park, Cardiff, CF14 4YS UK; 3 Department of Oncology and Metabolism, University of Sheffield Medical School, Beech Hill Road, Sheffield, S10 2RX UK; 4 Breast Unit, Royal Marsden NHS Foundation Trust, London, UK; 5 epiGenesys, Floor C, Cathedral Court, 1 Vicar Lane, Sheffield, UK; 6 Brighton and Sussex Medical School, University of Sussex, Falmer, Brighton, UK; 7 Department of Health Economics and Decision Science, School for Health and Related Research, University of Sheffield, Sheffield, UK; 8 Clinical Trials Research Unit, University of Sheffield, ScHARR, 30 Regent Street, Sheffield, UK; 9 Yorkshire and Humberside (formerly North Trent Cancer Network) Consumer Research Panel UK, Sheffield, UK

**Keywords:** Breast cancer, Older women, Decision support, Shared decision-making, Intervention implementation, Process evaluation

## Abstract

**Background:**

The Bridging the Age Gap in Breast Cancer research programme sought to improve treatment decision-making for older women with breast cancer by developing and testing, in a cluster randomised trial (*n* = 1339 patients), two decision support interventions (DESIs). Both DESIs were used in the intervention arm and each comprised an online risk prediction model, brief decision aid and information booklet. One DESI supported the decision to have either primary endocrine therapy (PET) or surgery with adjuvant therapies and the second supported the decision to have adjuvant chemotherapy after surgery or not.

**Methods:**

Sixteen sites were randomly selected to take part in the process evaluation. Multiple methods of data collection were used. Medical Research Council (MRC) guidelines for the evaluation of complex interventions were used.

**Results:**

Eighty-two patients, mean age 75.5 (range 70–93), provided data for the process evaluation. Seventy-three interviews were completed with patients. Ten clinicians from six intervention sites took part in telephone interviews. Dose: Ninety-one members of staff in the intervention arm received intervention training. Reach: The online tool was accessed on 324 occasions by 27 clinicians. Reasons for non-use of the online tool were commonly that the patient had already made a decision or that there was no online access in the clinic. Of the 32 women for whom there were data available, fifteen from the intervention arm and six from the usual care arm were offered a choice of treatment. Fidelity: Clinicians used the online tool in different ways, with some using it during the consultation and others checking the online survival estimates before the consultation. Adaptation: There was evidence of adaptation when using the DESIs. A lack of infrastructure, e.g. internet access, was a barrier to the use of the online tool. The brief decision aid was rarely used. Mediators: Shared decision-making: Most patients felt able to contribute to decision-making and expressed high levels of satisfaction with the process. Participants’ responses to intervention: Six patients reported the DESIs to be very useful, one somewhat useful and two moderately useful.

**Conclusions:**

Clinicians who participated were mainly supportive of the interventions and had attempted some adaptations to make the interventions applicable, but there were practical and engagement barriers that led to sub-optimal adoption in routine practice.

**Trial registration:**

ISRCTN46099296. Registered on 11 August 2016—retrospectively registered

**Supplementary Information:**

The online version contains supplementary material available at 10.1186/s13063-021-05360-z.

Contribution to the literature

- It is important to explore the implementation and mechanisms of impact of interventions to understand how the interventions may have led to the trial outcomes.
- Within this process evaluation of two decision support interventions, clinicians were mainly supportive of the interventions and had attempted some adaptations, but implementation of some elements of the interventions was limited.
- Practical and engagement barriers were found, supporting previous studies that demonstrate difficulties in implementing shared decision-making interventions.
- Team ‘buy in’ (“cognitive participation”) and practical facilitation (“collective action”) of the interventions could be targeted to improve implementation of these interventions into routine clinical practice.

## Background

The Bridging the Age Gap in Breast Cancer research programme sought to improve treatment decision-making for older women (≥ 70 years) with breast cancer by:

1. conducting an observational cohort study assessing breast cancer outcomes of older women,
2. developing decision support interventions (DESIs) to predict treatment outcomes and support shared decision-making,
3. testing the DESIs in a cluster randomised trial.

Two DESIs were developed and tested [1–3]. In frailer (defined as, those with decreased physiological reserve and increased vulnerability to negative health outcomes) older women (with oestrogen sensitive breast cancer), one DESI supported the decision to have either primary endocrine therapy (PET) or surgery with adjuvant therapies (standard care). In fitter older women with high recurrence risk breast cancer, the second DESI supported the decision to have adjuvant chemotherapy after surgery or not.

These treatment decisions are clinically important, having a substantial impact on cancer and treatment outcomes in older women [4–7]. Older women who have standard care for their breast cancer have improved overall and breast cancer-specific survival [3, 8] unless they are very frail, when surgery may cause significant harm [9, 10]. There are complex trade-offs to be made because standard care (including surgery for all and radiotherapy and chemotherapy if appropriate), whilst oncologically superior, is associated with negative quality-of-life impact and higher rates of complications [9, 11]. In women over the age of 70, there is uncertainty about the benefits of adjuvant chemotherapy due to the lack of clinical trial data specific to this older age group [12]. There is also evidence that rates of both surgery [9, 13] and chemotherapy [14] in this older age group vary widely both between units in the UK and across Europe [15, 16]. Decisions about treatment in these contexts can therefore be challenging both for clinicians (we use this term to include all types of healthcare professionals) and patients and are often “preference sensitive” [17]. Shared decision-making is particularly applicable in situations of preference sensitive treatment decisions and DESIs aim to support this process and improve the quality of treatment decisions.

### Summary of the trial

The trial was a multi-centre, parallel group, pragmatic, cluster randomised controlled trial (cRCT) nested within a larger cohort study of older women (> 70 years) with early breast cancer ('Age Gap Cohort Study’ (ISRCTN 46099296)) [18]. In December 2015, 46 of the 57 Age Gap cohort recruiting sites transitioned to a cRCT, with half of these sites randomised to use of the DESIs to support shared decision-making, and half to continue usual care. The primary outcome was improvement in quality-of-life. The findings from the cRCT are reported in a separate publication [19].

### The intervention

**Fig. 1:**
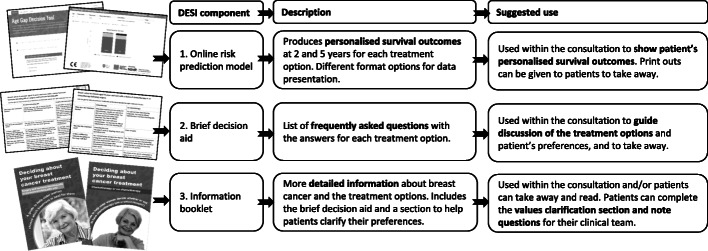
Details of the DESI components and suggested use; available at https://agegap.shef.ac.uk/

The DESIs were developed to ensure that the information contained was accurate, relevant and desired by the target population and written and presented optimally for women of an older age demographic [1, 20, 21]. Each DESI included three components: (1) an online risk prediction model [2, 3, 21], (2) a brief decision aid [1] and (3) an information booklet [1] (see Fig. 1). Component 1: The online tools were designed to help clinicians provide personalised survival predictions for each treatment option (PET versus surgery with endocrine therapy; or chemotherapy versus none after surgery). Clinicians could also print out these details for their patients. Component 2: The brief decision aids were designed to be used within a consultation as a visual tool to support shared decision-making. They contain frequently asked questions and answers for each treatment option. They aimed to help clinicians discuss the key information about the treatment options with their patients, and help patients think about what matters most to them about the treatment options and convey this to their clinician. Component 3: The information booklets provided more detailed information about the treatment options and included sections to help patients identify what mattered most to them about each treatment option. Further details can be found at https://agegap.shef.ac.uk/.

Staff in the intervention arm sites were given the DESIs to use as part of their usual clinical practice, regardless of whether patients took part in the study or not. Usual care sites were given information about the background to the study but no intervention training (or DESIs).

### Intervention training

Staff received training before implementing the intervention, and further support once implemented. Training highlighted the importance of attitudes and skills for shared decision-making, which are more important than the DESIs themselves [22]. The DESIs act as a tool to *support* shared decision-making [22]. As well as face-to-face training, there were three instructional screencasts and an animation available on the Age Gap website. Two screencasts showed how to use each of the online decision tools, i.e. for surgery with endocrine therapy or PET and surgery with or without chemotherapy, and one showed how the booklet and the brief decision aid were developed and how to use them in a consultation. These screencasts were designed to support the face-to-face training; the content covered was the same as in the face-to-face training. The animation demonstrated how the DESIs were *intended* to be used with patients in the Age Gap study, but that this was not prescriptive.

### Process evaluation

Alongside the trial a detailed process evaluation was undertaken. The Medical Research Council (MRC) guidelines for the evaluation of complex interventions were used to investigate the implementation and mechanisms of impact of the intervention to explore the process by which the intervention may have led to its effect or not [23]. The primary aims of the process evaluation were to understand how the DESIs were used/implemented, how acceptable and useful the DESIs were to both staff and patients, and barriers and facilitators to future implementation. We also sought to assess shared decision-making as a short-term process through which the interventions could improve quality-of-life by improving confidence in making the best decision and reducing regret about the choice made [18].

## Methods

### Regulatory approval

Ethics approval and research governance approval was obtained (IRAS: 12 LO 1808). All patients and clinicians gave written informed consent. The study was sponsored by Doncaster and Bassetlaw Teaching Hospitals NHS Foundation Trust.

### Sites and sample

Sixteen sites, eight from each arm of the trial, were randomly selected to take part in the process evaluation, stratified by whether sites had low or high rates of recruitment into the cohort study (Age Gap Cohort Study (ISRCTN 46099296)).

### Measures

Multiple methods of data collection were used to assess the implementation of the intervention and its mechanisms of impact. Implementation was assessed by examining **dose, reach, fidelity and adaptations** and mechanisms of impact were assessed by examining **mediators and participants’ responses** (see Table 1).

**Table 1:** Summary of the methods of data collection used in the Age Gap Trial process evaluation

Process evaluation assessment goal	Methods of data collection^**1**^	Description of data collected	Methods of analysis
**Implementation**			
**Dose:** *(Clinicians)* *"the quantity of intervention implemented."* [23]	Clinicians: website login	Record of the number of times the online tool was used by each clinician.	Descriptive statistics
**Reach:** (*Patients)* *"whether the intended audience [patients] comes into contact with the intervention, and how." *[23]	a) Bespoke patient completed questionnaire b) Patient semi-structured interviews c) Bespoke case report forms	Details of the information received, how it was used and how useful patients found it. (See supplementary file 3) Exploration of patients' perceptions of the information given, how it was used and satisfaction with decision-making. Details of how and when clinicians used the tools and reasons why they did not. (see supplementary files 1 and 2)	Descriptive statistics Framework approach Descriptive statistics
**Fidelity:** *(Clinicians and Patients)* "*whether the intervention was delivered as intended."* [23]	a) Clinician semi-structured interviews b) Patient semi-structured interviews c) Clinician website access to personalised risk sheet	Exploration of clinicians' views of the DESI, how it was used and their views of its usefulness. See above. Record of the number of times the personalised risk sheet was downloaded by each clinician.	Framework approach Framework approach Descriptive statistics
**Adaptations:** *(Clinicians)* *"alterations made to [the] intervention [by the clinician] in order to achieve better contextual fit."* [24]	Clinicians semi-structured interviews	See above.	Framework approach
**Mechanism of Impact**			
**Mediators:** "*intermediate processes which explain subsequent changes in outcomes."* [24]	a) CollaboRATE [25] b) Clinician semi-structured interviews c) Patient semi-structured interviews	Measure to assess the extent of shared decision-making. See above. See above.	Descriptive statistics Framework approach
**Participants’ (patients’) responses to intervention:** *"Participant responses to and interactions with the intervention"* [23]	a) Bespoke patient completed questionnaire b) Patient semi-structured interviews	See above. See above.	Descriptive statistics Framework approach

^1^Data were not collected from clinicians at usual care sites

To assess **dose** (to clinicians) and **reach** (to patients), quantitative data from the intervention online tool log, trial case report forms (see supplementary files 1 and 2) and a bespoke (developed in-house) (*Discussing treatment options*; see supplementary file 3) patient questionnaire were collected. Semi-structured interviews were also used to ascertain dose and reach.

To assess **fidelity, adaptation** and **participants’ responses to the DESI**, we investigated how the interventions were used and how acceptable and useful they were. This was examined both from clinician (adaptation) and patient (participants’ responses) points of view, primarily using semi-structured interviews, but also using a bespoke patient questionnaire. The CollaboRATE questionnaire [25] was used to assess the extent of shared decision-making as a potential **mediator** of the effect of the DESIs. For the purposes of this study, we have assessed **adaptation** as alterations made to the implementation of the DESIs by clinicians and **participants’ responses** were assessed by the patients' reactions to the intervention.

### Implementation guidance

Clinicians were encouraged to use the DESIs to support usual practice, but we were not highly prescriptive in how this should be done. The training materials included an animation example of how the DESIs might be used to encourage shared decision-making. It was acknowledged that this may not always be possible. Clinicians were encouraged to use one or more of the three elements of the relevant DESI during discussions about treatment (see Fig. 1).

### Procedure

#### Patients

Patient participants were recruited if they were willing to be contacted about optional ‘further’ components of the trial (ascertained in the main trial consent form) and had had a discussion either about PET or surgery with endocrine therapy, or about chemotherapy. Eligible patients were provided with an invitation pack (letter, information sheet, study reply form and reply envelope). Patients who returned a completed study reply were contacted to discuss any questions and to arrange a face-to-face interview. Consent for the interview was taken at the time of the interview.

Participants were sent the 6-week process evaluation follow-up questionnaire when timing allowed. CollaboRATE was collected as part of the main trial baseline questionnaire.

#### Clinicians

Clinicians at intervention sites who could be involved in a consultation to discuss treatment options were invited to take part. Invitation packs (letter, information sheet, consent form and reply envelope) were given to local research staff to distribute to relevant clinical staff. Consent forms included consent for participation in an interview.

### Data analysis

Descriptive statistics were used to analyse the questionnaires, case report forms and website log data. CollaboRATE was scored using the CollaboRATE Top Score method favoured for its ease of interpretation. Scores range between 0 and 100 with higher scores representing greater shared decision-making [25].

Patient and clinician interviews were audio-recorded, pseudo-anonymised and transcribed verbatim. Data were analysed using a thematic approach and organised using the principles of the framework approach [26]. Initial interview transcripts were read by two researchers (KL and MB) discussed and codes agreed before developing an analytical framework which was then applied to the remaining interviews using NVivo 11 and charts were then created using Microsoft Excel. Key categories were identified using both a deductive approach, guided by the topics in the interview guide, and inductive approach, based on the data. Codes relevant to the aims of the process evaluation were selected and analysed to describe the themes in the data pertaining to reach, fidelity and the mechanism of impact, i.e. mediators and the participants' responses to the intervention (see Table 2).

**Table 2:** Patient interview themes

Final themes	Codes	MRC framework items mapped to codes
**Treatment choice and decision-making**	Treatment choice and decision	Reach, fidelity
	Feelings about decision	
	Feelings about how decision was made	Mediators
**Use and usefulness of information**	What information was received	Reach
	How information/DESI was used	Fidelity, participant response to intervention
	Perceived usefulness of information	Participant response to intervention
**Impact of information**	Impact of information/DESI/discussion	Participant response to intervention

## Results

### Patient sample

The sample was derived from 1339 trial participants, of whom 495 were from process evaluation sites. Of these, 324 patients indicated on the trial consent form that they would be happy to be approached for involvement in other parts of the study and 165 patients were invited to take part.

A total of 84 patients agreed to take part; however, two patients withdrew. A total of 82 patients, median age 75 (range 70–93), provided data although they did not take part in every aspect of the process evaluation.

Seventy-three patient interviews were completed (see Table 3). Of these, 35 were from intervention sites and 38 from usual care sites.

**Table 3:** Data collected and participant characteristics

	Intervention arm	Usual care arm
**Patient interviews (*n*** **= 73)**	*n* = 35	*n* = 38
Median (range) age in years	76 (70–88)	73.5 (70–93)
Mean age in years	76.4	74.8
**Questionnaires completed**:		
CollaboRATE **(*n*** **= 18)**	*n* = 9	*n* = 9
Median (range) age in years	76.0 (72–83)	74 (72–93)
Mean age in years	76.0	75.7
Bespoke questionnaire **(*n*** **= 24)**	*n* = 11	*n* = 13
Median (range) age in years	75.0 (70–79)	74 (70–93)
Mean age in years	74.2	75.6

### Clinician sample

Ten clinicians from six intervention sites took part in telephone interviews. This included eight surgeons, one oncologist and one nurse practitioner.

### DESI implementation

#### “Dose”: take up of staff training

**Fig. 2:**
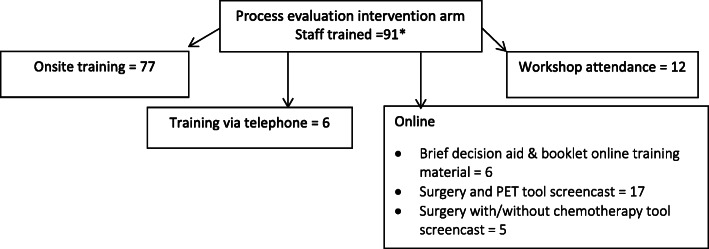
Flowchart to show number of staff trained and training mode. Asterisk indicates that some participants received additional telephone training

Ninety-one members of staff from process evaluation sites in the trial’s intervention arm received intervention training (see Table 4). Of these, 12 attended a standardised offsite workshop, 77 attended onsite customised training sessions, and six received telephone instruction (see Fig. 2; some staff attended more than one training session). The amount of time spent in training varied considerably. Workshops ran for approximately 120 min, whilst the customised on-site training time ranged from 15 to 120 min with a median of 60 min.

**Table 4:** Training received by staff in process evaluation sites, split by staff group

	Training attendees
**Surgeons/physicians**	21
Admin	12
**Clinical nurses**	19
Research nurses	14
**Oncology medics**	6
Trials staff	4
Imaging staff	6
Miscellaneous	8
Unknown	1
**Total**	**91**

#### “Reach”: use of DESIs with patients

The online tool data showed that the tool was accessed on 324 occasions, from all eight process evaluation sites by 27 clinicians (Table 5). It was not possible to tell whether this was as part of training or contact with a patient.

**Table 5:** Use of online tool

	Number of times online tool accessed		Number of times personalised risk sheet downloaded
Process evaluation sites (*n* = 8)	324	282 PET/surgery with endocrine therapy 42 chemotherapy	107 (from 5 sites; 3 sites did not download personalised sheets at all)

All eight intervention sites accessed and ran the online tool to varying degrees. One site accessed it on only one occasion whilst others ranged from 18 to 80 occasions (median 45). Not all chose to download the personalised sheet even though they had accessed the tool (Table 5).

Of the 82 patients who provided data in the process evaluation, 41 (50%) were reported on the case report form to have had a consultation in which either PET or surgery with endocrine therapy (*n*=26, 31.7%) or chemotherapy (*n*=15, 18.3%) was discussed. Six (7.3%) had a consultation about both, 20 (24.4%) had neither and case report forms were not completed for the remaining 15 (18.3%) patients. See Table 6 for details.

**Table 6:** Numbers of patients from the case report form relating to patient choice with each treatment choice in process evaluation study sites

		Usual Care	Intervention	Total
		***N*** = 44	***N*** = 38	***N*** = 82
Consultation for:	Both	2 (4.5%)	4 (10.5%)	6 (7.3%)
	Surgery with endocrine therapy or PET only	10 (22.7%)	16 (42.1%)	26 (31.7%)
	Chemotherapy only	11 (25.0%)	4 (10.5%)	15 (18.3%)
	Neither	16 (36.4%)	4 (10.5%)	20 (24.4%)
	Missing	5 (11.4%)	10 (26.3%)	15 (18.3%)

Data from the case report forms show 15 patients from intervention sites were offered a choice of PET or surgery with endocrine therapy and five were not offered a choice. Two of the 15 offered PET or surgery with endocrine therapy were also offered chemotherapy. Six other patients were offered a choice about chemotherapy. In the usual care arm, six patients were offered a choice of PET or surgery with endocrine therapy and six were not. Two patients were offered a choice of both PET or surgery with endocrine therapy and chemotherapy, and eight others were offered a choice about chemotherapy.

**Fig. 3:**
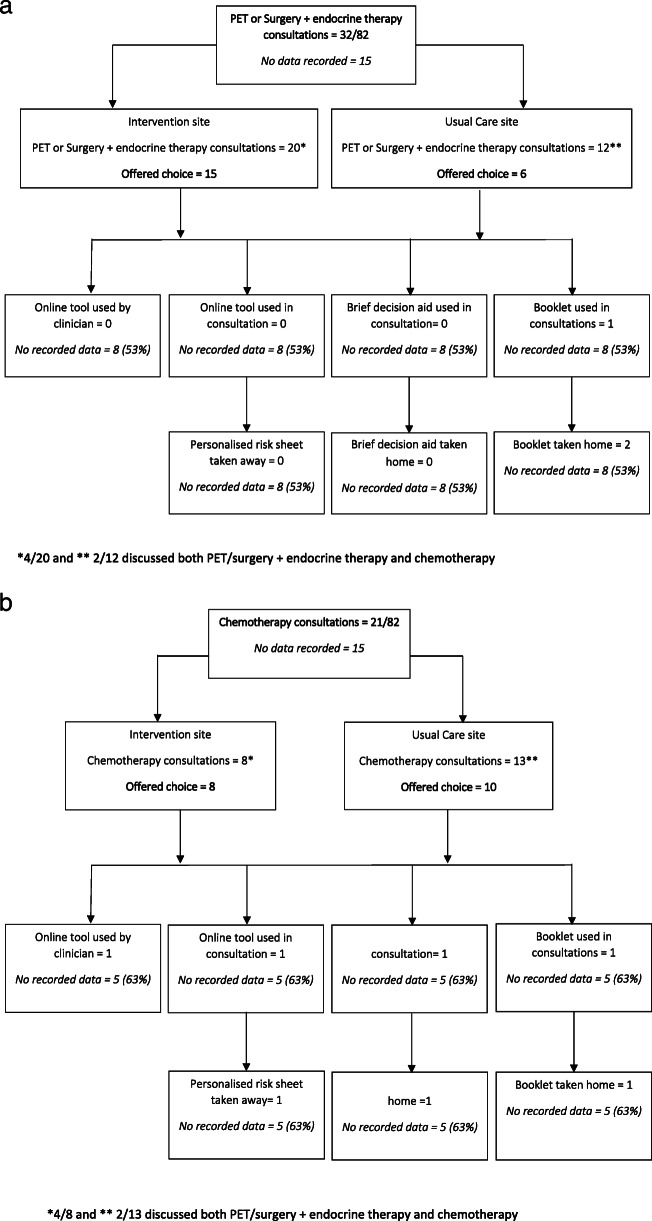
**a** Flowchart to show *reach* of DESI for PET or surgery with endocrine therapy in patients offered a choice of treatment. **b** Flowchart to show *reach* of DESI for chemotherapy in patients offered a choice of treatment

For the 15 with a choice of PET or surgery with endocrine therapy in the intervention arm, clinicians did not report use of the online tool. It was used once to aid a chemotherapy decision. The personalised sheet was given to one chemotherapy patient but not given to any of the PET or surgery with endocrine therapy patients (see Fig. 3a and b). The top two reasons for non-use of the online tool were that the patient had already made a decision or that there was no online access in the clinic. In one case, it was felt that the chemotherapy patient was unsuitable. The brief decision aid was used once during a chemotherapy consultation and one chemotherapy patient was given it to take away. Reasons for non-use of the brief decision aid were the same as for to the online tool. The booklet was used once during a PET or surgery consultation and once in a chemotherapy consultation, these patients were given it to take away and one patient was given the PET or surgery with endocrine therapy to take away (see Fig. 3b). Reasons for non-use were again that the patient had already made their treatment decision or that the booklets were not available in the clinic. Completion of the case report form to record DESI usage was incomplete with over 50% of the data missing.

During interviews, although all women remembered receiving information, only some (18/35) of the women recalled specifically receiving any of the DESI materials. Furthermore, women reported receiving different elements of the DESI: ten of the 35 remembered the booklet, five the brief decision aid and three the personalised sheet. Only one chemotherapy patient remembered all elements of the DESI.

#### “Fidelity”: intervention delivered as intended

Most of the clinicians interviewed were surgeons and had mainly used the PET/surgery with endocrine therapy DESI. All the surgeons interviewed had used the online tool. The oncologist interviewed did not have access to the online tool due to technical issues within the Trust but was keen to use it when it was made available to her. Some of the surgeons reported that they had used the booklets and/or brief decision aids, but they also reported that they were aware that they were used by the nurses with patients. Although the nurse practitioner had not used the DESIs, she had observed consultants and nurses using elements with patients during consultations and offering them to patients to take away.

Clinicians reported using the online tool in two different ways. Some went through the inputs (patient characteristics) and outputs (treatment dependent survival predictions) with the patient, and then offered or sent the printout to patients. Others looked at the survival predictions before the consultation. Survival estimates calculated by the online tool were seen by the surgeons as an objective basis upon which to base discussions of the treatment options.

Clinicians also used the booklets and brief decision aids in different ways with some going through them during the consultation, others giving them to patients to take away and others not using the DESIs. Findings from both the patient and clinician interviews most frequently found the brief decision aids were only rarely used to support treatment choice discussion as intended but were more usually given as a summary of the treatment options.

“Yeah, the [pause] with regards to the booklet, and the question and answer sheet (PET v Surgery brief decision aid), I’ve sort of briefly described what that’s about to the patient, but in general I will give it to them to look at in their own time.” (Breast surgeon)

“I: And do you remember...how it (PET v Surgery brief decision aid) was used?

P: I think the doctor went through the options with me and then I was given one of those to bring home.” (78 years, breast-conserving surgery)

Some patients reported being given or sent the brief decision aid and/or booklet to read at home, for others the personalised print out and/or booklet were discussed and used during the consultation and for one woman the brief decision aid was used during the consultation. Very few women used the values clarification exercise because they felt a decision had already been made (either by themselves or through recommendation from the clinician).

#### “Adaptation”: to show how the intervention was tailored

From the clinician interviews, there was evidence of adaptation of using each of the DESIs. Although all three elements of the intervention were available for patients, it was common for them to be given only one, usually the booklet. Where the brief decision aid was used, it was almost exclusively given as a means of providing a summary of information and not as a prompt to engage the patient in discussion before decision-making.

Some clinicians felt that providing the survival outcomes information calculated by the online decision tool was too “harsh” to give to the patient when the survival predictions were poor. In this situation, clinicians moderated this information and gave only the 2-year survival predictions or explained the differences in survival between treatments rather than showing the absolute values. (In designing the tools, this was the reason for including 2 year outcomes so women with very short life expectancy due to extreme old age or ill health would be able to see some survival data).

#### Barriers and facilitators to use

During interviews, clinicians concentrated on the usefulness of the online tools and commented on how they found these helped the discussion with patients. Clinicians found the survival predictions of the online tool useful to inform their own clinical judgement about treatment, as well as their patients’ opinions. They liked that this was evidenced-based, with objective figures they could present to their patients and sometimes the families. One surgeon had found it particularly helpful in reassuring a patient and her family that surgery was not the best option.

However, comments were made by clinicians about the current lack of validation of the data supporting the online tools and they felt this may be a barrier to use (validation had been performed but not published when the trial commenced). For some, the logistics of using the tool(s) within the consultation were a problem. The lack of infrastructure, e.g. computers and printers within the clinics, and the need for internet access were all cited as barriers. Some felt that using the online tools and/or the booklet and brief decision aid was, however, potentially feasible within a consultation and was largely down to personal organisation. However, one surgeon felt it was not possible within the clinic appointment duration. Some clinicians felt using the online tool during the Multi-Disciplinary Team (MDT) meeting would have been beneficial, but the logistics of having patient information and computers available were again cited as barriers. (However, many MDTs do now have access to online algorithms during the MDT and use of PREDICT is now commonplace so this should not be a problem going forward.)

Clinicians felt that more detailed training in the use of DESIs would allow them to be more effectively integrated into their practice. During the course of the trial, many sites had staff changes which led to poor consistency and loss of expertise in using the DESIs. This was cited as a barrier to implementation.

Clinicians commented that they felt the information provided from the DESI elements gave the patient confidence to engage in the decision-making process and feel more content with their treatment decision. This had encouraged some of these clinicians to continue using the DESI tools. Some surgeons welcomed how the DESI had changed the dynamic of the consultation and had also made them more open-minded in considering treatment options.

Those clinicians who used the booklets and brief decision aids commented that it was ‘custom and practice’ for the patients to be given large quantities of cancer-related information (e.g. from breast cancer charities—see below) and that the DESI information was likely to be lost in all this. This was cited as a potential barrier to implementation.

### DESI mechanisms

#### “Mediators”: shared decision-making

Across both arms of the trial, most women reported being offered treatment options and were satisfied with the way the decision was made. Many said they felt involved in the decision-making process. Some stated specifically that the information they received had given them the confidence and knowledge to be involved. Women spoke highly of clinicians: how they had given them time and information, been supportive and listened to their preferences. This view was further supported by the results of the CollaboRATE questionnaire. Eighteen patients completed a CollaboRATE questionnaire, nine in each trial arm. All scored very highly, with the intervention score range from 90 to 100 and usual care 93 to 100, demonstrating high levels of satisfaction.

#### “Participants’ responses to intervention”

Eleven patients from the intervention arm completed the bespoke questionnaire and seven reported the DESIs to be very useful, one somewhat useful and two moderately useful (one response was missing).

Women were given a vast quantity of generic breast cancer information and this meant the DESIs were often lost amongst it all. National cancer charities produce comprehensive and appealing looking folders and generic treatment booklets which are easy for clinicians to distribute but overwhelmed some patients who felt that the information was irrelevant for them (not age or disease specific for them). Many women reported finding the information they received about their treatment useful, though not expressly the DESIs. This usefulness was predominantly to do with knowing what the treatment would involve, being guided through the process and receiving confirmation of information also given verbally by the clinicians. They felt the information enabled them to more fully engage in the decision-making process. They could understand what was being or had been discussed and the information gave reassurance about making the right decision. For some, the combination of the different information formats allowed them to feel involved in the decision-making process. For instance, some patients found that the written information prompted further questions which they raised during subsequent consultations.

Some women who were considering chemotherapy and were shown output from an online tool (it was unclear whether this was the Age Gap tool or other tool such as PREDICT) and used this information to either accept or reject the option of chemotherapy. Only one woman was sure it was the Age Gap online tool she had seen. However, during the interviews it became clear that not all the women understood the output from the tool despite using the information to inform their decision.

Patients made largely favourable comments about the layout and presentation of all the information received. Two women interviewed felt they received too much irrelevant, general information, not specifically the DESI, and this was a barrier to using the information.

## Discussion

This study examined the implementation of two DESIs to support older women with breast cancer treatment decisions. Despite the high number of patients recruited to the cRCT, the reported implementation of some elements of the DESIs was limited at the intervention sites participating in this process evaluation. Most women reported receiving a lot of information about treatments, both written and verbal. Generic (non-trial specific) written information included booklets produced by charities, locally provided leaflets as well as the trial specific DESI materials. Some women described information as being irrelevant which further justified our development of age specific informational resources for this study. Some women from intervention sites had not received any elements of the DESI. The DESIs received positive feedback from the clinicians interviewed, in particular the online component, which is supported by the higher usage than is reported in the case report forms. However, clinicians reported less use of the booklets and brief decision aids, with some suggesting that perhaps the breast nurses or research staff used these or provided them to patients. Patient experiences of discussing treatment options varied, both depending on their own case (what options were available) and on the consulting clinician. Most women felt involved in the decision-making process, but the degree to which this was true varied between individuals; some reported being given a choice of treatments, whereas others reported a recommended treatment plan.

This process evaluation was based on a randomly selected sample of participating study sites, stratified for recruitment rates into the cohort study as a measure of research engagement. Mixed-methods data collection was used, comprising web-log data, training participation, questionnaires and case report forms, and parallel interviews with both patients and clinicians (surgeons, nurses, oncologists). However, data capture and participation were also incomplete, highlighting some limitations. Firstly, with regard to data capture, completion of the case report forms documenting use of the DESIs was poor with often more than half of report forms uncompleted. During patient interviews, it was difficult to know whether they were commenting on the DESIs or on other information they received (e.g. from breast cancer charities), thus providing a degree of uncertainty in our findings. Secondly with regard to participation, there is potential bias in the clinician sample particularly, as a number of clinicians did not volunteer to participate, and non-responders may view the DESIs less favourably. Thirdly, regarding both data capture and participation, the small amount of data relating to the chemotherapy decision points to the need for further investigation particularly around decision-making for this treatment option. A further limitation is that this evaluation was part of a large-scale research trial, and the findings may have limited transferability for implementation efforts in routine practice [27].

However, the findings are consistent with other experiences nationally and internationally about the difficulties of implementing shared decision-making interventions [22, 28]. The DESIs investigated here were rigorously developed with patient needs assessment, field testing and clinician and patient input to consider their “fit” as supportive interventions for normal practice. Although the trial showed limited impact of the DESIs on quality-of-life (the primary end point of the trial [19]), they did impact on treatment choice and knowledge scores. The process evaluation elements (italicised below) enable us to identify where further intervention is required if greater effects in improving quality-of-life and quality of shared decision-making are to be achieved. This can also be interpreted in relation to Normalisation Process Theory [29] (the core components are in quotation marks below) about adoption of technology or interventions into practice, as has been undertaken in relation to shared decision-making previously [30, 31].

There was good participation in training and in the small number interviewed, favourable attitudes to the purpose and elements of the DESIs, indicating reasonable *dose* of the intervention and “coherence” or sense making of the participants regarding implementation. The exception to this coherence was one site that did not use any aspect of the DESI following training. There were however significant practical barriers, such as the lack of online access in clinics and printer availability, as well as the extent of other information also available to patients, causing information overload and distraction from the DESIs. These challenges impacted the *fidelity* of the interventions, indicating problems in the clinical teams which maybe explained with reference to two core components, “cognitive participation” and “collective action”, of the Normalisation Process Theory [29].

Cognitive participation is described as the “relational work”, which in this study can be interpreted as the clinical team’s initiation, organisation or re-organisation of working practices (getting ‘buy in’), engagement with and belief that staff can make a valid contribution to the new practice. There was evidence of teams continuing with their usual decision-making practices and making little attempt to adjust to incorporate the DESIs.

Collective action is the “operational work” that people do to endorse a complex intervention in everyday practice. It describes how they are facilitated to implement and how they use and experience the intervention. It was clear that sites differed in the way that they allocated resources and responsibility to the delivery of the intervention with some making the research staff alone responsible for the use of the DESIs and not implementing them as part of usual practice as intended. Turnover of team members often interrupted the implementation. Practical challenges were identified which need attention and support, including efforts to resolve online access and printer availability. There was evidence of *adaptations* made by clinicians, such as selecting which survival data were felt most applicable, indicating consideration of the “workability” of the intervention. Clinicians reported that the use of the DESIs, most frequently the PET or surgery with endocrine therapy, was associated with shared decision-making and thus is a *mediator* and indicates the inconsistent way staff used the intervention in everyday practice (“contextual integration”). Skill development for shared decision-making, through enhanced training, may be an area that can be strengthened in future implementation efforts, to enhance both “cognitive participation” and “collective action” towards making these interventions more “normal” in practice. We found some evidence of favourable *responses* from patients, when the DESIs were used. Formal appraisal and feedback, i.e. “reflexive monitoring”, to clinicians was not part of the complex intervention and thus could be strengthened also in future implementation efforts.

Further research is required regarding effective implementation strategies for DESIs, particularly for older patient groups. Specific interventions such as shared decision-making skill development require evaluation. Implementation work requires intensive support [22]. It will also be important to explore how to engage and support the clinical team better in identifying ways of enhancing the “cognitive participation” and “collective action” amongst team members, to achieve more successful implementation in practice.

## Conclusion

Whilst preliminary results suggest positive feedback about the DESIs, from both patients and clinicians, limited use is also apparent from our data. Clinicians were mainly supportive of the interventions and had attempted some adaptations to make the interventions more applicable. However, there were still considerable practical barriers that led to limited adoption of the interventions in routine practice. These barriers need to be addressed if older patients are to be supported to make effective decisions about important health care conditions and to achieve best outcomes in their personal situation.

## Supplementary Information

**Additional file 1.** Case report form—treatment decision support consultations.

**Additional file 2.** Case report form—treatment decision.

**Additional file 3.** Bespoke questionnaire—discussing treatment options.

## Data Availability

Part of the data generated or analysed during this study is included or is available from the corresponding author on reasonable request.

## Abbreviations

cRCT: Cluster randomised controlled trial
DESI: Decision support intervention
MDT: Multi-disciplinary team
NIHR: National Institute for Health Research
PET: Primary endocrine therapy
