# Supplementary material for: Process evaluation of the Bridging the Age Gap in Breast Cancer decision support intervention cluster randomised trial
Source: Trials. 2021 Jul 13;22:447. doi: 10.1186/s13063-021-05360-z (PMC8278730; doi:10.1186/s13063-021-05360-z)
Supplement: Supplementary file 1 — Additional file 1. Case report form—treatment decision support consultations. [file 13063_2021_5360_MOESM1_ESM.pdf]

# Age Gap

Baseline (RCT)

FOR OFFICE USE ONLY

Enrolment No. E ☐☐ / ☐☐☐

## Treatment decision support consultations

To be completed by the treating clinician or clinical nurse specialist as soon as possible after the treatment decision support consultation(s) took place.

**Was there a consultation about treatment with primary endocrine therapy or surgery and adjuvant endocrine therapy?**

☐ Yes ☐ No

Date of consultation ☐☐ ☐☐ ☐☐☐☐
d d m m y y y y

Was the patient offered a choice between primary endocrine therapy or surgery and adjuvant endocrine therapy? ☐ Yes ☐ No

Did the patient follow the recommended treatment? ☐ Yes ☐ No

*Following this consultation, please:*

- *complete the* ***Treatment decision*** *form*
- *provide the participant with the relevant* ***Treatment options and decision questionnaire*** if they:
  - are a full participant
  - were offered a choice between primary endocrine therapy or surgery and adjuvant endocrine therapy
  - have not already completed it in relation to a choice between chemotherapy or no chemotherapy

**Was there a consultation about whether or not to have chemotherapy?**

☐ Yes ☐ No

Date of consultation ☐☐ ☐☐ ☐☐☐☐
d d m m y y y y

Was the patient offered a choice between chemotherapy or no chemotherapy? ☐ Yes ☐ No

Did the patient follow the recommended treatment? ☐ Yes ☐ No

*Following this consultation, please:*

- *complete the* ***Treatment decision*** *form*
- *provide the participant with the relevant* ***Treatment options and decision questionnaire*** if they:
  - are a full participant
  - were offered a choice between chemotherapy or no chemotherapy
  - have not already completed it in relation to a choice between primary endocrine therapy or surgery and adjuvant endocrine therapy
