# Supplementary material for: Process evaluation of the Bridging the Age Gap in Breast Cancer decision support intervention cluster randomised trial
Source: Trials. 2021 Jul 13;22:447. doi: 10.1186/s13063-021-05360-z (PMC8278730; doi:10.1186/s13063-021-05360-z)
Supplement: Supplementary file 2 — Additional file 2. Case report form—treatment decision. [file 13063_2021_5360_MOESM2_ESM.pdf]

Age Gap

Baseline (RCT)

FOR OFFICE USE ONLY

Enrolment No. E _ _ / _ _ _

Treatment decision

To be completed by the treating clinician or clinical nurse specialist following the patient's treatment consultation.

1. Which treatment options were discussed?
(***tick one only*** *– complete an additional form if necessary)*

☐ Chemotherapy or no chemotherapy

☐ Primary endocrine therapy or surgery and adjuvant endocrine therapy

2. Which components of the intervention tools were used and how?

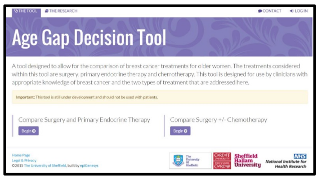

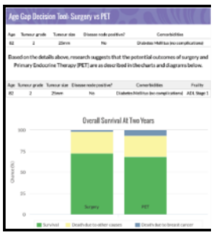

a. Age Gap Online Decision Tool

i. Used by the clinical staff to aid their decision making ☐ Yes ☐ No

Tick all that apply

☐ Not enough time
☐ Patient not felt suitable
☐ Not liked by clinical staff
☐ No online access in clinic
☐ Patient already made a decision
☐ Other____________________

ii. Shown to the patient and discussed in the presence of the doctor or clinical nurse specialist ☐ Yes ☐ No

Tick all that apply

☐ Not enough time
☐ Patient not felt suitable
☐ Not liked by clinical staff
☐ No online access in clinic
☐ Other____________________
☐ Patient too distressed
☐ Patient unable to understand
☐ Patient already made a decision
☐ Family/carer reluctance

iii. Print out taken home by patient ☐ Yes ☐ No

Tick all that apply

☐ Not enough time
☐ Patient not felt suitable
☐ Not liked by clinical staff
☐ No online access in clinic
☐ Not offered
☐ Other____________________
☐ Patient too distressed
☐ Patient unable to understand
☐ Patient already made a decision
☐ Patient reluctance
☐ Family/carer reluctance

Age Gap

Baseline (RCT)

FOR OFFICE USE ONLY

Enrolment No. E ☐☐ / ☐☐☐

Treatment decision

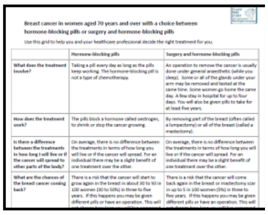

b. Option grid

i. Used within the consultation ☐ Yes ☐ No

Tick all that apply

- ☐ Not enough time
- ☐ Patient not felt suitable
- ☐ Not liked by clinical staff
- ☐ Not available in clinic
- ☐ Other____________________
- ☐ Patient too distressed
- ☐ Patient unable to understand
- ☐ Patient already made a decision
- ☐ Patient reluctance
- ☐ Family/carer reluctance

ii. Taken home by patient ☐ Yes ☐ No

Tick all that apply

- ☐ Not enough time
- ☐ Patient not felt suitable
- ☐ Not liked by clinical staff
- ☐ Not available in clinic
- ☐ Not offered
- ☐ Other____________________
- ☐ Patient too distressed
- ☐ Patient unable to understand
- ☐ Patient already made a decision
- ☐ Patient reluctance
- ☐ Family/carer reluctance

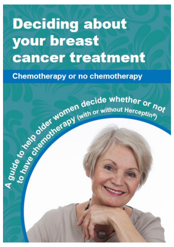

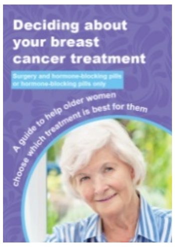

c. Booklet "Deciding about your breast cancer treatment"

i. Used within the consultation ☐ Yes ☐ No

Tick all that apply

- ☐ Not enough time
- ☐ Patient not felt suitable
- ☐ Not liked by clinical staff
- ☐ Not available in clinic
- ☐ Other____________________
- ☐ Patient too distressed
- ☐ Patient unable to understand
- ☐ Patient already made a decision
- ☐ Patient reluctance
- ☐ Family/carer reluctance

ii. Taken home by patient ☐ Yes ☐ No

Tick all that apply

- ☐ Not enough time
- ☐ Patient not felt suitable
- ☐ Not liked by clinical staff
- ☐ Not available in clinic
- ☐ Not offered
- ☐ Other____________________
- ☐ Patient too distressed
- ☐ Patient unable to understand
- ☐ Patient already made a decision
- ☐ Patient reluctance
- ☐ Family/carer reluctance
