# Supplementary material for: Process evaluation of the Bridging the Age Gap in Breast Cancer decision support intervention cluster randomised trial
Source: Trials. 2021 Jul 13;22:447. doi: 10.1186/s13063-021-05360-z (PMC8278730; doi:10.1186/s13063-021-05360-z)
Supplement: Supplementary file 3 — Additional file 3. Bespoke questionnaire—discussing treatment options. [file 13063_2021_5360_MOESM3_ESM.pdf]

Section 2 Discussing treatment options

E [ ][ ][ ] / [ ][ ][ ]
FOR OFFICE USE ONLY

These questions are for the 'Discussing treatment options' part of the Bridging the Age Gap in Breast Cancer study. Please complete them if you wish and send this sheet back in the freepost envelope.

We would like to ask you some questions about being given the treatment options of surgery and hormone-blocking pills or hormone-blocking pills only.

Date of completion [ ][ ] [ ][ ] [ ][ ][ ][ ]
d d m m y y y y

1. Who spoke to you about your treatment options?

Please tick all that apply

- ☐ The doctor from the breast unit
- ☐ The nurse from the breast unit
- ☐ A doctor or nurse or similar on a telephone helpline
- ☐ GP or practice nurse
- ☐ Other (please say who) ____________________

2. Were you provided with any booklets/leaflets about breast cancer and/or your breast cancer treatment options?

☐ Yes ☐ No

A 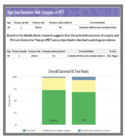

B 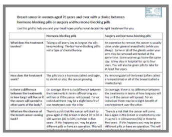

C 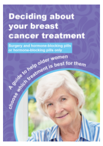

If yes, which of the following?

Please tick all that apply

- ☐ Personalised risk information (print out from the doctor/nurse's computer) [A]
- ☐ Option grid [B]
- ☐ Information booklet called "Deciding about your breast cancer treatment" [C]
- ☐ Other (please give details) ____________________

3. If you were given information to take away how did you use it?

Please tick all that apply

- ☐ I read it all
- ☐ I read some of it
- ☐ I did not read it
- ☐ I showed it to friends or family
- ☐ I filled in some or all of the blank sections in the booklet
- ☐ Other (please give details) ____________________

4. Overall, how useful did you find the information about your treatment options in thinking about and making your decision (if you had a choice)?

- ☐ Not at all useful
- ☐ Somewhat useful
- ☐ Moderately useful
- ☐ Very useful

5. **How useful did you find the different parts of the information about your treatment options in thinking about and making your decision (if you had a choice)?**

a) **Personalised risk information (print out from the doctor/nurse's computer)**

I did not receive this or did not read or use it	Not at all useful	Somewhat useful	Moderately useful	Very useful
☐	☐	☐	☐	☐

b) **Option grid (or "options at a glance")**

I did not receive this or did not read or use it	Not at all useful	Somewhat useful	Moderately useful	Very useful
☐	☐	☐	☐	☐

c) **"Deciding about your breast cancer treatment" booklet <u>information sections</u> (e.g. about breast cancer, surgery, hormone-blocking pills)**

I did not receive this or did not read or use it	Not at all useful	Somewhat useful	Moderately useful	Very useful
☐	☐	☐	☐	☐

d) **"Deciding about your breast cancer treatment" booklet <u>"My decision" section</u> (e.g. my questions, weighing up my options)**

I did not receive this or did not read or use it	Not at all useful	Somewhat useful	Moderately useful	Very useful
☐	☐	☐	☐	☐

6. **What did you think about the amount of information you were given?**

☐ Not enough
☐ About right
☐ Too much
